# Recent Stem-Cell-Based and Stem-Cell-Free Possibilities for the Therapeutic Management of the Osteonecrosis of the Jaw

**DOI:** 10.3390/biom15040595

**Published:** 2025-04-16

**Authors:** Merita Mazreku, L’uboš Danišovič, Martin Klein, Mária Kleinová

**Affiliations:** 1 Institute of Medical Biology, Genetics and Clinical Genetics, Faculty of Medicine, Comenius University, Sasinkova 4, 811 08 Bratislava, Slovakia; mazreku1@uniba.sk (M.M.); lubos.danisovic@fmed.uniba.sk (L.D.); 2 Institute of Histology and Embryology, Faculty of Medicine, Comenius University, Sasinkova 4, 811 08 Bratislava, Slovakia; maria.kleinova@fmed.uniba.sk

**Keywords:** osteonecrosis of the jaw, stem-cell-based therapy, exosomes, tissue engineering

## Abstract

Osteonecrosis of the jaw (ONJ), including the maxilla and mandible, is considered a challenging therapeutic problem, mainly due to the lack of understanding of its pathogenesis. It is well known that ONJ is a severe side effect caused by certain medications used to treat bone metastasis and osteoporosis, such as bisphosphonates, which inhibit bone resorption. Other therapeutics with similar side effects are, for instance, receptor activators of nuclear factor kappa-B ligand (RANK-L) inhibitor (denosumab), tyrosine kinase inhibitors (sunitinib), and antiangiogenics (bevacizumab). The conservative or surgical treatment of these medication-related osteonecroses of the jaw (MRONJs) is generally effortful and still not entirely effective. Therefore, the research seeks alternative treatment options like tissue engineering and stem cell therapy, which predominantly represent mesenchymal stem cells (MSCs) and their derivatives, such as extracellular vesicles. Moreover, it was published that novel stem cell therapy could even prevent the onset of MRONJ. On the other hand, the administration of stem cells may also be accompanied by some other health risks, such as an increased chance of cancer metastasis occurrence in cancer patients. The current review paper summarizes the most recent progress in stem-cell-based and stem-cell-free treatment options for the ONJ. Similarly, we discuss this novel approach’s future perspectives and possible obstacles.

## 1. Introduction

Osteonecrosis of the jaw (ONJ) is a condition most often caused by drugs like antiresorptive agents, for instance, bisphosphonates (BPs), but also receptor activators of nuclear factor kappa-B ligand (RANK-L) inhibitors, like denosumab, and antiangiogenic drugs, like bevacizumab. This condition is known as medication-related osteonecrosis of the jaw (MRONJ). There are also other identified causes like radiation therapy (osteoradionecrosis), trauma-induced osteonecrosis, and non-traumatic and spontaneous osteonecrosis [1]. ONJ is characterized by progressive bone damage in the maxillofacial region, which can lead to chronic pain syndrome, infection, and disfigurement [2]. MRONJ most often develops due to several precipitating factors. These factors include tooth extraction, which is by far the most common, accounting for about 60% of cases [3], followed by ill-fitting dentures [4], oral surgical intervention, and periodontitis [5]. A significant proportion of the cases, around 15%, can occur spontaneously [6].

The incidence has not been well established until recently. In 2024, Brunner et al. [7] published a population-based multicenter retrospective study that evaluated breast cancer patients in nine breast centers in Austria between 2000 and 2020. The incidence of MRONJ was 11.6% in the denosumab-only cohort, 2.8% in the BP group, and 16.3% in the group taking both medications in succession. Focusing more on the Pan-European epidemiological evaluation, Boffano et al. [8] analyzed data from different clinics in Italy, Serbia, Spain, France, Denmark, Hungary, Slovenia, Czech Republic, Croatia, Greece, Poland, Lithuania, and Bulgaria. The studied period was from 2013 to 2022. The indication for antiresorptive or antiangiogenic mediation was either osteoporosis or bone metastasis. Although the authors did not report the overall incidence of MRONJ, they focused on various characteristics of patients affected by the condition. For instance, the study showed that MRONJ is more typical in older women (mean age 71 years), and there is a strong association between MRONJ risk and pharmacotherapy length and dosage. More than 90% of patients are treated surgically; however, in 15.3% of cases there are postoperative complications, and in 20.8% of patients reoperation is needed.

The epidemiological data and the drawbacks of conventional treatment strategies make the novel therapeutic options urgent. This review focuses on the state-of-the-art therapeutic options for MRONJ using individualized stem-cell-based and stem-cell-free therapies.

## 2. The Pathophysiology of MRONJ

**Figure 1:**
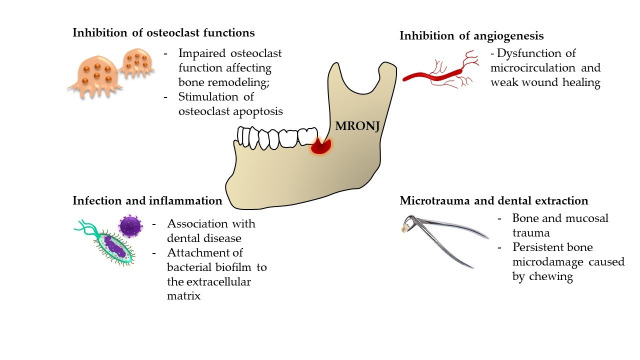
Principal pathophysiological mechanisms of MRONJ.

The pathophysiology of MRONJ is complex, with different pathophysiological phenomena at play, depending on the causative agent (Figure 1). BPs are most commonly prescribed for osteopenia and osteoporosis treatment due to their ability to inactivate osteoclasts [9]. Other indications include Paget’s disease, different neoplasms, and neoplasm-associated conditions [10]. In general terms, oncological patients are one of the most affected groups, since these agents are indicated for the management of cancer-related conditions, including malignancy-associated hypercalcemia, skeletal-related events associated with bone metastases in the context of solid tumors such as breast cancer, prostate cancer, and lung cancers as well as for management of lytic lesions in the setting of multiple myeloma. In 2021, Teoh et al. [11] analyzed the Database of Adverse Event Notification from the Therapeutic Goods Administration in Australia and found out that from the 419 identified case reports of MRONJ, 405 were associated with BPs and denosumab. Only 14 cases were caused by secondary agents. The first case of MRONJ caused by prolonged BP use was first described in 2003. Marx [12] published a letter to the editor addressing a “growing epidemic” of avascular necrosis caused by pamidronate and zoledronate. Since BPs were the only known cause of this condition, bisphosphonate-related ONJ (BRONJ) was the term of choice. Only after the other medications mentioned above were described in the etiopathogenesis did MRONJ become the preferred term. Even though the pathophysiological mechanisms had been assumed correctly based on the known actions of BPs on osteoclast physiology (inhibition of monocyte differentiation into osteoclasts, stimulation of osteoclast apoptosis, or reduction of osteoclast activity), the exact cause of MRONJ was elusive. In 2022, almost 20 years after the first description, Okawa et al. [13] concluded that the pathogenesis of MRONJ is still far from established.

Since the jawbone is closely related to the oral cavity, and BPs, in most cases, affect the jawbone exclusively, the authors hypothesized that specific immunological cross talk between oral mucosa and underlying bone plays a significant role. Investigating this notion, Elsayed et al. [14] hypothesized that zoledronate binds to hydroxyapatite in the bone extracellular matrix (ECM), enhancing bacterial biofilm attachment to the ECM. The authors found that after hydroxyapatite discs were treated with zoledronate, the biofilm attachment was more significant than in the control group. Another step of the experiment was the application of the oral biofilm to a model of extraoral bone defect on a rat tibia. The result was osteonecrosis, which indicated that zoledronate binds to ECM hydroxyapatite and is in close contact with the oral cavity’s unique bacterial makeup, making the jawbone prone to MRONJ [14]. There are several other particular characteristics of the maxilla and mandible that are unlike those found in skeletal structures of different locations, making them susceptible to osteonecrosis, but also other conditions affecting the jaw exclusively, like cherubism [15] or hyperparathyroid jaw tumor syndrome [16]. The first characteristic is persistent bone microdamage caused by chewing and local inflammation, which exposes the bone to harmful pathogens. When this scenario is combined with suppressed bone remodeling due to the discussed medications, bone death may ensue. This etiopathogenic framework is called an “inside-outside” hypothesis.

**Figure 2:**
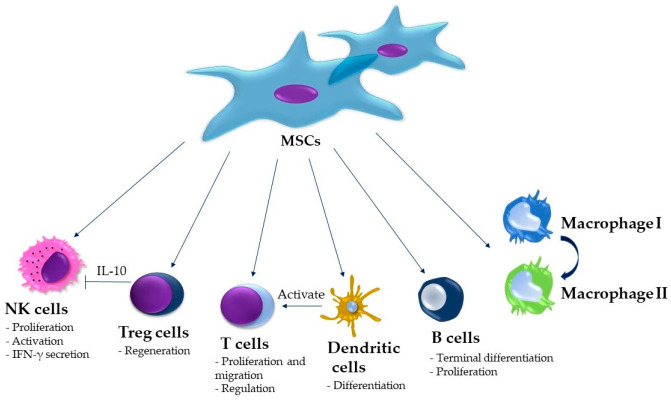
Possible MSC effects on different immune cell populations, addressing the MRONJ-associated immune dysregulation.

The second hypothesis is called “outside-inside”, which postulates that the medications cause local immune suppression, which makes it challenging for the oral mucosa microenvironment to fight off pathogens breaching the mucosal barrier, which eventually leads to the spread of pathogens to the underlying bone [17]. This immune suppression is mediated, e.g., through BP-induced dendritic cell dysfunction, which causes harmful bacterial overgrowth and impaired T-cell response [18]. All this is possible since the jawbones and oral cavity are separated only by thin mucoperiosteum, making them very weakly protected compared to other bones in the human body, which are separated from the outside environment by a thick layer of skin and muscle [19]. Dendritic cells are not the only immune cells at play, though. The immune dysfunction can be seen as a common denominator for different pathogenetic mechanisms, eventually leading to MRONJ. The drugs themselves modify the immune response by stimulating the production of proinflammatory mediators. When damage occurs, e.g., due to complicated tooth extraction, T cells release IL-17, which supports the differentiation and proliferation of osteoblasts, leading to bone healing and repair. However, this proinflammatory cytokine must be regulated before an overkill reaction causes more harm than good. When this inhibition does not occur, IL-17 overaction can have the opposite effect on osteoblasts and stimulate osteoclasts, leading to bone resorption [20]. Macrophages are another leukocyte population that plays an important role in MRONJ pathogenesis. In a recent paper, Soundia et al. [21] reported that M1 polarization of macrophages associated with the overexpression MMP-13 has a crucial role in the early stages of MRONJ. Another player is NK cells, which communicate with osteoclasts in a significant manner. Namely, NK cells are, under physiological conditions, able to act as cytotoxic factors toward osteoclasts, hindering their bone-resorbing action. In 2015, Tseng et al. [22] experimentally demonstrated that BP-treated osteoclasts became resistant to NK-induced cytotoxicity. Finally, some lines of research indicate that some species of oral bacteria can directly regulate the RANKL expression in B cells, promoting further bone resorption in the already-disrupted bone and periodontal microenvironment by the aforementioned immune dysregulation [23]. All these factors produce a pathological setup, predisposing the jawbones toward MRONJ. As we will discuss in greater detail in the following paragraphs, different aspects of these immune dysregulations can be successfully tackled by MSC-based therapies, leading to optimistic future therapeutic outlooks (Figure 2).

The diagnostic criteria for MRONJ fall under three broad categories, which can be assessed based on history and clinical examination. First is the history of the current or past administration of BPs, RANK-L inhibitors, or antiangiogenic drugs. Secondly, upon clinical examination, a physician observes bone exposure or bone that can be reached through fistulas in the maxillofacial region present for at least the past 8 weeks. Finally, there cannot be a positive history of metastasis of the jawbone or radiation exposure in this region [24]. Based on the classification criteria of the American Association of Oral and Maxillofacial Surgeons, MRONJs can be divided into three stages plus the zero stage. No clinical presentation of bone necrosis defines stage 0, but there are some clinical signs and symptoms with low specificity and minor radiographic indicators. In stage 1, there is a visible necrotic bone or fistula, but the patient has no symptoms or signs of infection. To be classified as stage 2, there must be a clinically manifested infection with pain and redness, with or without pus secretion. Finally, stage 3 is assessed when there are one or more of the following findings present on top of the criteria necessary for stage 2: necrotic bone extends beyond alveolar bone, there is a pathologic fracture, extraoral fistula, oral antral/oral nasal communication, or finally, there is osteolysis that extends to the inferior border of the lower jaw or the sinus floor [25].

## 3. MRONJ—Current Therapeutic Options

A 2021 meta-analysis discussed therapeutic options for MRONJ in general. Goker et al. [26] published a systematic review of 118 papers. By far, the most common treatment option was a surgical intervention found in 75 papers, followed by platelet concentrate in 15, laser therapy in 10, conservative protocols in 9, parathormone-derived molecule teriparatide in 4, hyperbaric oxygen in 3, and finally, ozone application in 2 papers. All reported positive outcomes. Multiple meta-analyses investigated and compared studies that focused on individual therapeutic strategies aimed to mitigate MRONJ; aside from the mentioned teriparatide therapy [27] and autologous platelet concentrates [28,29], other approaches were thoroughly analyzed, including human amniotic membrane [30] and laser surgery [31,32]. Despite numerous advantages, all these approaches have multiple disadvantages, mainly in terms of efficacy, the invasive nature of surgical procedures, the risk of recurrence, and incomplete functional restoration. Therapy of MRONJ is also a challenge because there is no consensus in the scientific community and clinicians on which therapeutic option is the most optimal. Moreover, there is a substantial heterogeneity in patient response to available therapies, underscoring the necessity of a personalized approach [33].

## 4. Stem-Cell-Based Therapy of the MRONJ

Due to the above-mentioned hurdles in MRONJ’s therapeutic management, the scientific community has started to seek a solution for alternative forms of treatment, such as the application of stem cells. Several research papers have referred to the positive effect of stem cells on wound healing in MRONJ animal models (Table 1) and patients by restoring immunological abnormalities and reducing inflammatory cytokines [34,35,36,37]. Moreover, recent studies investigated the impact of adipose-tissue-derived stem cells (ADSCs) on preventing the onset of MRONJ [38,39].

The highly used stem cell type in clinical trials in various diseases related to the bone or cartilage are MSCs, thanks to their multipotency reflected in the ability to give rise to tissue-forming cells, such as osteoblasts and chondroblasts. Moreover, their immunomodulatory properties make them a good candidate for bone grafting material in case of osteonecrosis. The secretome of MSCs, specifically exosomes, also contains essential molecules involved in cellular processes, such as apoptosis, migration, proliferation, and specific differentiation. Thanks to this particular cargo, the MSC-derived exosomes can control or induce bone, cartilage, or dentin tissue formation [47,48,49,50].

MSCs have been used in MRONJ, primarily in the form of cell grafts. Several authors have reported positive outcomes of MSC grafts in pigs, mice, and humans. For instance, Kikuiri et al. [40] developed a mouse-MRONJ disease-like model via intravenous zoledronate or dexamethasone administration for 2 to 7 weeks following tooth extraction. The mentioned drugs caused the onset of disease symptoms by suppressing adaptive regulatory T-cells and Tregs and activating Th17. These MRONJ animal models recapitulated the chief disease symptoms, including an open alveolar socket, exposed necrotic bone, or increased inflammation. Therefore, they represented a fitting disease model for stem cell therapy. The MSCs were isolated from C57BL/6J mice’s bone marrow and infused via the tail vein. After administering MSCs for two weeks, the inhibition of Th17 and increased restoration of Treg levels were observed, resulting in positive treatment outcomes, such as complete mucosal healing and bone regeneration. Therefore, according to the authors, the crucial mechanism of MRONJ pathogenesis is to reveal the immunologic balance between Treg/Th17 cells. Moreover, the researchers stated that systemic MSC treatment can prevent MRONJ development. Published results represented the first evidence of successful immunotherapy with MSCs in MRONJ-like mice. From that time, several papers were published describing the efficient outcome of MSCs in MRONJ treatment; however, the unsolved part was the instability of MSCs. In some cases, the instability of MSCs and their hyperactive coagulation can lead to the circulation of cells throughout the body, causing pulmonary embolism or the death of animal models. Moreover, in cancer patients, the intravenous (IV) infusion of MSCs can increase the proliferation of cancer cells [51,52]. Therefore, the intravenous administration of MSCs is almost impossible in clinical applications. Hence, because of the instability of MSCs, Kaibuchi et al. [37] developed a tissue-engineered cell sheet of allogenic MSCs and transplanted it to the MRONJ rat model. The results showed better wound healing in the MSC sheet group than in the control and MSC intravenous injection groups. More concretely, active neoangiogenesis, a necessary step in tissue regeneration, was observed in models treated with MSC sheets. The process of neoangiogenesis was proved by the presence of vascular endothelial growth factor and hepatocyte growth factor in the supernatant secretion. According to the immunohistochemical analysis, the authors observed the differentiation of EGFP-positive MSCs into pericytes and undifferentiated MSCs in the wound healing area two weeks after the transplantation. Based on the research group’s experience with the treatment of MRONJ since 2013, the authors, in their latest study, announced an interest in conducting a clinical trial of stem-cell-based therapy for MRONJ using periodontal-ligament-derived MSC sheets [53].

A research group led by Zang et al. [39] published results that proved that the local transplantation of ADSCs on tooth extraction in BP-treated animals prevents the development of BRONJ. Firstly, the authors induced the MRONJ-like lesion in a rabbit model via administering zoledronic acid (ZA) and dexamethasone weekly during the 8 weeks. After the following premolar extractions, ADSCs incubated on coral hydroxyapatite were placed into the extraction sockets of animal models. The tissue samples from sockets were harvested for further analysis, such as flow cytometry, immunochemistry, TGF-β1 ELISA, and real-time PCR in weeks 2 and 8. Two weeks post-extraction, the primary gingival wound healing was observed, and the inflammatory infiltration in the connective tissue under the socket was much smaller than in the control group. Similarly, the proportion of collagen deposition in the subgingival connective tissue was significantly higher in the ADSC group; moreover, at the transplantation site, many osteoclasts reflected the bone remodeling process. A comparable outcome was also observed at 8 weeks post extraction, involving a lower proportion of necrotic bone, higher activity in new bone formation, and significant healing of the gingival epithelium. The results from quantitative PCR and immunohistochemistry showed that behind the wound-healing mechanism in the ADSC group is a higher expression of TGF-β1 and fibronectin. In particular, the increased TGF-β1 levels influenced by the presence of ADSCs were found to be a critical factor in preventing BRONJ onset by promoting early gingival healing and isolating the wound microenvironment from the rest of the oral cavity. Generally, it is known that TGF-β1 cytokine is involved in epithelial healing and facilitates the promotion of granulation tissue formation and re-epithelization [54,55,56].

An interesting study was published by Barba-Recreo et al. [42] in which the investigators treated ZA-induced MRONJ rat models with a combination of ATSCs and platelet-rich plasma (PRP). PRP is hypothesized to have a positive regenerative effect, especially in wound healing; therefore, its combination with stem-cell-based therapy could be even more beneficial. The authors of the above-mentioned study investigated the effect of different mixtures of ATSCs and PRP local applications on wounded alveolar sockets. Results showed no macroscopically evident alveolar bone exposure. Moreover, osteonecrosis was significantly reduced. Intense vascularization, higher osteoclast number, and bone remodeling were detected in ATSCs and ATSCs + PRP groups. According to the authors, the combination of ATSCs and PRP appeared to be synergic.

Alonso-Rodriguez et al. [44] investigated the therapeutic response of ADSCs on BRONJ rat models. BRONJ was induced in 38 Wistar rats by intraperitoneal ZA injection. After the teeth were extracted, the animals in the control group received saline solution over the alveolar socket, and the animals in the treatment group received ADSCs instead of saline solution. An absorbable hemostatic gelatin sponge was used as a carrier for saline solution or ADSCs. The histological analysis was performed at 1 and 2 months after teeth extraction. Regarding the osteonecrosis of the alveolar bone, both groups displayed similar distributions of osteonecrotic bone tissue; however, in the ADSC-treated group, significantly higher new bone formation and vascularization were observed. Other features, such as inflammatory infiltrates, number of osteoclasts, and bone remodeling, did not show differences between control and ADSC-treated groups.

The research group led by Rodríguez-Lozano et al. [45] examined the effect of allogeneic bone marrow (BM)-MSC transplantation on the MRONJ Wistar rat models. The MRONJ in rat models was induced by ZA administration. The first group of MRONJ rat models also received 1 × 10^6^ allogeneic BM-MSCs seeded on a synthetic β-tricalcium phosphate (β-TCP) construct, which serves as a bone graft substitute. The second group was the control group, which received only saline solution on the β-TCP construct. In both groups, three upper molars were extracted in the eighth week of treatment. Four weeks after teeth extraction, the wound sites in each animal were macroscopically and histologically analyzed. Subsequently, several other analyses were performed, such as immunohistochemistry and PCR. Macroscopically, there was no evidence of exposed bone or uncovered alveolar socket typical for MRONJ in any of the animals in group 1, compared to 33% of the animals in group 2 that displayed osteonecrotic lesions in the teeth extraction sites. The histology of maxillary bone from animals in the control group revealed typical histopathologic features of osteonecrosis, such as inflammatory cell infiltrates, fibrosis, and granulation tissue formation. On the other hand, samples made from the maxillary bone from the first group of animals treated with BM-MSCs showed new bone formation accompanied by increased osteoblasts and osteoclasts. This was also proved by the expression of osteocalcin-positive cells, which was significantly higher in the BM-MSC-treated group, reflecting the intense process of bone neoformation. Taken together, the authors proved that the transplantation of allogeneic BM-MSCs on a proper scaffold material at the tooth extraction site can represent an effective alternative for improving MRONJ management or preventing its development.

Likewise, BRONJ large animal models (minipigs) were treated with allogeneic BM-MSCs in the study published by Li et al. [41]. The results of the BM-MSCs treatment were also enthusiastic, considering mucosal healing and bone reconstruction, as well as a decrease of proinflammatory IL-17 levels and elevation of Tregs. However, the BM-MSCs were administered via IV infusion. According to the authors, the BM-MSCs are capable of homing the wounded bone tissue, even with intravenous delivery, and have an anti-inflammatory effect.

The preventive impact lying behind the mechanism of applying ADSCs in the treatment of MRONJ was most recently studied by Dong et al. [38]. The study was conducted on MRONJ mouse models treated by daily intraperitoneal injection of ZA to induce osteonecrosis. The maxillary first molar extraction was performed after 2 weeks, and animals were randomly divided into three groups: the control group, the MRONJ group, and the ADSC group. Immediately after teeth extraction, animals in the ADSCs group received intravenously the ZA and ADSCs, which were resuspended in the phosphate-buffered saline solution. One week post-extraction, the ADSC group exhibited faster gingival epithelium healing and lower bone exposure rate than the MRONJ group. Histological analysis further confirmed the efficacy of ADSCs. As mentioned above, one of the reported triggers of MRONJ is also dental implant surgery and peri-implantitis, which can lead to chronic inflammation and progressive loss of supportive bone [4,57]. Based on that, the research team of Nishimaki et al. [46] described a new rat model of MRONJ created by subcutaneous administration of zoledronate and dexamethasone and subsequent placement of titanium materials (screw and a plate) on the buccal side of the mandibular bone into the pre-drilling hole. Thereafter, one group of animals, the MSC sheet + group, received two grafts of MSC sheets placed directly onto the titan plates and covered with soft tissue. The MSC sheets were formed from allogenic BM-MSCs isolated from rats’ femurs and tibias. Six weeks after the surgery, mandibular bones were removed, and titanium materials were further examined. Histological examination of MSC sheet (+) and MSC sheet (–) groups showed empty lacunae, suggesting the necrotic area; however, the bones from the MSC (+) group also contained normal lacunae adjacent to the empty ones. Generally, the accumulation of empty lacunae was near the titanium implant, assuming the connection between the implant and the absence of osteocytes. In the MSC (+) group, new laminar bone formation around the titanium materials was observed. Despite the limited number of animals, the published results suggest that it may help cover titanium reconstruction materials with MSC sheets in complicated cases of alveolar bone reconstruction.

Worth mentioning is also the MRONJ treatment strategy based on the use of stromal vascular fraction (SVF) cells. It is known that SVF obtained by enzymatic treatment and centrifugation of adipose tissue has regenerative potential similar to stem-cell-based therapy, thanks to different cell content (adipocytes, hematopoietic stem cells, endothelial cells, stromal cells) [58]. For instance, Kuroshima et al. [43] examined the therapeutic effect of noncultured SVF cells on the MRONJ mice model induced by ZA and cyclophosphamide (CY) treatment after tooth extractions. SVF cells were isolated from mouse fat pads and further processed. Subsequently, the noncultured SVF cells were transplanted via tail-vein injection. After 4 weeks, the euthanasia of the mice models was performed to investigate the osseous and soft maxillary tissue changes. The SVF treatment results were very encouraging compared to the control group in the sense of wound healing, increased number of osteoclasts and osteocytes, and significantly decreased necrotic bone area. Therefore, the use of SVF cells should be considered as a treatment option for MRONJ.

Almost all published studies used MRONJ animal models, and only a few researchers investigated the effect of MSCs application on humans affected by MRONJ. A study published by Elad et al. [59] was among the first pioneering papers reporting the application of allogenic MSCs on the margins of the exposed bone of MRONJ patients. The authors recorded complete wound healing in 5 months. Another groundbreaking study was published by Cella et al. [60]. It was a case report in which complete lesion healing was achieved in a patient with MRONJ stage 3 after applying autologous BM-MSCs. The patient was a 75-year-old woman undergoing treatment with alendronate and pamidronate due to severe osteoporosis. The conservative, non-surgical treatment of developed MRONJ was ineffective. Therefore, the research team performed autologous BM-MSCs transplantation into the mandibular lesion. The autologous BM-MSCs were harvested from the posterior superior iliac crest by aspiration into heparinized syringes and further processed for transplantation. A fibrin sponge and an activated platelet-rich plasma suspension were carriers for the patient’s BM-MSCs. One week after the surgical procedure, the carrier was removed. The improvement of the lesion accompanied by symptom reduction was observed two weeks post-surgery. A patient showed significant progress in bone healing and concentric ossification during the following six-month period. A complete healing of MRONJ was achieved 30 months later.

Following case report studies, stem cell administration on patients with different MRONJ stages achieved similar healing results [61,62]. For instance, Voss et al. [61] investigated the stem-cell-based management of six patients with diagnosed MRONJ. Patients were undergoing treatment either with BPs due to osteoporosis or denosumab for breast cancer, and present in all of them was an exposed bone or extraoral fistula in the maxillofacial region for longer than 8 weeks. A periosteoplasty was performed for all patients to remove the affected necrotic bone region. Subsequently, patients’ BM-MSCs and autologous thrombin and collagen membrane were transplanted into the bone defect. The surgical wound was closed by a modified three-layered technique. Patients were clinically and radiographically examined during the follow-up period ranging from 12 to 54 months to evaluate the wound-healing process. The results of the cell-based treatment were very encouraging, involving complete mucosal healing without signs of infection or bone exposure in the surgical region. None of the other patients had developed any signs of recurrences of MRONJ, and there was no metastatic disease of the mandible.

More recently, De Santis et al. [62] reported two cases of MRONJ patients who were successfully treated by autologous BM-MSCs application. The first case was a 68-year-old man treated with cyclophosphamide, bortezomib, and dexamethasone for multiple myeloma. Moreover, a patient received several doses of ZA for bone disease. Later, the dental implant installation was performed. After that, the patient’s symptoms worsened, including constant local pain and several episodes of local mandibular infection resulting in MRONJ stage 2 development. The patient did not respond to conservative treatment; therefore, the authors decided to implant BM-MSCs locally at the side of the lesion. BM-MSCs were harvested from the posterior iliac crest and then underwent subsequent steps to achieve the final product suitable for transplantation. The BM-MSCs were first embedded into a small piece of bone substitute and thereafter transplanted after necrotic bone and dental implant removal. In addition, BM-MCS solution was injected around the bone substitute to boost the therapeutic effect of stem cells. The computerized tomography performed 6 months after the surgery displayed complete wound healing and proper bone regeneration. The patient also did not complain of any pain or inflammation. The second case represented a 66-year-old woman undergoing therapy with ZA due to metastatic breast cancer. After the treatment, the patient was diagnosed with MRONJ stage 2, accompanied by symptoms such as pain, local swelling, and pus exudation. Due to the very weak response of patients to conservative treatment, the authors decided to proceed with cell-based therapy. The procedure for obtaining BM-MSCs was the same as in the first case. The results of the treatment were very similar to those of previous therapies. Six months after the surgery, almost complete bone regeneration was observed, and the patient no longer complained of local pain or inflammation.

Another recent paper investigating the regenerative potential of adipose tissue SVT together with leukocyte-platelet-rich fibrin (L-PRF) for MRONJ treatment was published by Bouland et al. [63]. The authors reported two cases of ZA-induced MRONJ patients (stage II and III) treated with tissue-engineered L-PRF scaffold containing SVF cells accompanied by wound site closure with a mucoperiosteal flap. In both cases, buccal mucosal healing was achieved two weeks after the procedure, bone formation with osteocondensation was documented by three consecutive cone beam computed tomography studies, bony bridges were observed 18 months after the intervention, and no signs of clinical recurrence were seen during 18 months of follow-up. These preliminary results demonstrate the beneficial influence of MSCs associated with L-PRF-SVF.

All mentioned case reports have proved the importance of cell-based therapy in bone tissue regeneration and remodeling in patients suffering from MRONJ or even in preventing MRONJ development (Table 2). Various growth factors secreted by MSCs are particularly involved in bone healing, such as bone morphogenic proteins, insulin-like growth factors 1 and 2, transforming growth factor-b1, fibroblast growth factor 2, and platelet-derived growth factor. Therefore, the local application of BM-MSCs or ADSCs should become one of the therapeutic options for treating bone defects [64,65].

At the same time, before the large-scale clinical use of MSCs, there is a need to solve some issues related to the manner of their acquisition, usually not a comfortable invasive needle aspiration, followed by cultivation, purification, and calculation of the final concentration. Brozovich et al. [66] recently pointed out the high variability between bone marrow aspirate and bone marrow concentrate; therefore, the presence of MSC-specific types, concentration, and density must be determined. Moreover, it is essential to note that via needle aspiration, malignant cells could be aspired and then transplanted together with MSCs, which could potentially cause micrometastases to spread in the affected region. Another issue is choosing a proper MSC carrier with fitting properties for small bone lesions. The scaffold should support cell adhesion, osteoblastic growth, and differentiation of MSCs.

## 5. Stem-Cell-Free Therapy of MRONJ—Exosomes

At the same time, the paracrine secretion of MSCs is gaining increasing importance because of the secretome’s content, which involves at least 10 proteins responsible for tissue regeneration, osteogenesis, angiogenesis, and tissue regeneration. Exosomes are key paracrine modulators carrying this essential material [67].

Exosomes belong to extracellular vesicles that play a crucial role in intercellular communication. The vesicles exhibit high heterogeneity in morphology, with most of them being in the shape of hemispherical structures with clearly defined lipid bilayers, as observed using electron microscopy. They are formed via the endosomal pathway, originating from multivesicular bodies that fuse with the plasma membrane, whereby exosomes are released into the extracellular environment [68,69]. The molecular composition of exosomes is highly heterogeneous and differs according to the cellular origin of secretion. Exosomes transport numerous bioactive molecules that encompass small RNA types such as microRNAs (miRNAs), long non-coding RNAs (lncRNAs), circular RNAs, messenger RNAs, and an array of functional proteins. The lipid bilayer of exosomes serves as a protective shield, preserving the stability of the cargo in transit to the recipient [70].

One of the most significant characteristics of exosomes is their protein composition, which includes a variety of functionally important classes of proteins. These include ubiquitously occurring annexins in membrane structure and intercellular membrane fusion. Exosomes are also packed with RAS-related GTP-binding proteins, which have been shown to play a crucial role in intracellular vesicular transport. Exosomes also contain protein kinases and heterotrimeric G proteins, which control signal transduction pathways [71,72].

Furthermore, exosomes are also enriched with members of the tetraspanin superfamily, such as CD9, CD63, CD81, and CD82. Tetraspanins mediate the targeting of exosomes to target cells by interacting with integrins and major histocompatibility complex molecules, controlling immune responses. Aside from proteins, exosomes carry lipids such as cholesterol, sphingomyelin, and ceramides, responsible for structural integrity and stability in extracellular fluids [72].

Due to biogenesis, exosomes take on the molecular fingerprint of the original cell and therefore can serve as biomarkers for health and disease conditions. Their ability to transmit molecular messages between distant cells has increased the interest in their role in disease progression, immune response, and treatment. Of note is that exosomes carrying cell-type-specific molecular content, i.e., exosomes obtained under different conditions or from different tissues, may have distinct molecular content and activities. Being of very sophisticated composition and involved in the mediation of intercellular communication, exosomes are being increasingly viewed as a unique tool in multiple biological processes, from immune modulation and tissue repair to disease pathogenesis. Their therapeutic application in drug targeting and regenerative medicine is being intensely sought after, making exosomes up-and-coming candidates for the next generation of biomedical therapies [68].

The exosome-based therapies aid bone regeneration by delivering bioactive molecules such as miRNAs and growth factors, directly impacting osteogenic and osteoclast activity. ATSC-derived exosomes have the potential to promote bone remodeling and enhance soft tissue wound healing and angiogenesis [73]. Moreover, Watanabe et al. [74] reported the administration of MSC-derived exosomes, preventing the senescence of cells involved in wound healing and the spread of chronic inflammation around senescent cells, promoting angiogenesis and bone regeneration and preventing MRONJ.

Recent studies suggest the involvement of exosomes in the pathophysiology of MRONJ and their possible therapeutic effect. Exosomes influence MRONJ pathogenesis by modulating immune responses, mainly by inhibiting M1 macrophage polarization, reducing inflammation, and enhancing osteoblast activity [75]. Additionally, exosomes regulate T-cell responses by altering dendritic cell activity, suppressing overactive immune responses that contribute to the exacerbation of MRONJ. Kawai et al. [76] confirmed the regenerative effect of exosomes presented in conditioned media isolated from rat mesenchymal stromal cells cultivated with human umbilical vein endothelial cells and implanted into the periodontal defect. Two weeks after exosome implantation, bone regeneration was evident. Other analyses revealed various processes stimulated by MSC-derived exosomes, such as the mobilization of endogenous MSCs, angiogenesis, and differentiation. Even more, implanted exosomes boosted stem cell differentiation into the osteoblastic lineage, such as osteoblast or cementoblasts, giving rise to the new alveolar bone, cementum, and periodontal ligament. The authors concluded that the application of MSC-derived exosomes enormously affected bone regeneration.

Exosomes are also involved in osteoclast regulation. Osteoclast dysfunction is central to ONJ development, as BPs and denosumab inhibit bone resorption, leading to an accumulation of necrotic bone. Exosomes influence osteoclastogenesis through their cargo of miRNAs, lncRNAs, and signaling proteins. Specific miRNAs, such as miR-146a-5p and miR-322-3p, found in exosomes derived from bone marrow macrophages and osteoclast progenitors, have been shown to inhibit osteoclast differentiation, leading to decreased bone turnover [77]. Conversely, exosomes from adipose-derived MSCs have been shown to restore osteoclast–osteoblast homeostasis by reducing osteoclastic activity while stimulating osteoblast proliferation.

Studies show that exosomes derived from adipose-derived mesenchymal stromal cells prevent MRONJ progression by suppressing inflammatory mediators such as IL-1RA and promoting angiogenic factors [75,78]. More concretely, Zheng et al. [75] examined the effect of ADSC-derived exosomes on the MRONJ mouse model. The administration of ZA and subsequent tooth extraction established the MRONJ animal model. Exosomes were mixed with hydrogel and injected directly into the site of extraction. According to performed analyses such as micro-CT and histology, the tooth socket displayed full mucosa coverage, epithelial migration, and bone formation. Going deeper, the authors hypothesized that macrophage pyroptosis is one of the mechanisms behind slower wound healing in MRONJ patients.

Furthermore, exosome-based therapies aid bone regeneration by delivering bioactive molecules such as miRNAs and growth factors, directly impacting osteogenic and osteoclastic activity [77].

In regenerative medicine, exosome-based therapy is a new and promising approach to treating MRONJ. It is a non-invasive, cell-free modality versus MSC therapy, keeping immune-related risks in check [79]. Using exosomes as therapeutic agents and biomarkers highlights their potential in tailored medicine for ONJ [80].

## 6. Conclusions and Future Expectations

It is clear that with the increasing cases of MRONJ worldwide, the need for efficient treatment pressures researchers to seek novel therapeutic ways. One of them represents stem-cell-based therapy. The MSCs involving BM-MSCs and ADSCs are primarily favorable, thanks to their ability to differentiate osteogenic, chondrogenic, and adipogenic lineages. Moreover, they possess anti-inflammatory and immunoregulatory capacities mediated by paracrine secretion. Also, MSCs have been intensively studied in many clinical applications, such as musculoskeletal system regeneration, wound healing, angiogenesis, and even nerve regeneration [81,82]. Therefore, it was predictable that researchers would try to use MSCs as an alternative to the conservative treatment of MRONJ.

According to published papers, stem-cell-based therapy for MRONJ can become a more successful treatment. However, the number of MRONJ case reports with positive outcomes of stem-cell-based treatment is still very low; therefore, further studies are needed to clarify their full use in clinical practice. At the same time, it is inevitable to standardize protocols regarding the number of cells, the best type of MSCs, and the suitable method of MSC administration.

Meanwhile, novel therapeutic options represented by cell-free methods using extracellular vesicles, concretely exosomes, have been spotlighted. Exosome treatment may become a new strategy for various diseases thanks to their capability to carry almost all therapeutic effects of MSCs, such as anti-inflammatory activities and tissue regeneration potential. On the other hand, they minimize the safety concerns related to MSC injection and are more easily handled, characterized, and stored. Therefore, MCS-derived exosomes have tremendous potential as therapeutic agents.

## Figures and Tables

**Table 1:** Selected MRONJ-induced animal models.

Animal Model	Pre-Treatment	Type of MSCs	Administration	Outcome	Ref.
Mouse	IV ZA and dexamethasone	BM-MSCs from C57BL/6J mice	Infusion via tail vein	- Complete mucosal healing and bone regeneration- The inhibition of Th17 and restoration of Treg levels	[40]
Minipig	IV ZA	Allogeneic BM-MSCs	Intravenous infusion	- Mucosal healing- Bone reconstruction- Decrease of proinflammatory IL-17 levels and elevation of Tregs	[41]
Rat	IV ZA	Allogenic ADSCs/PRP	Direct placement into the socket	- No bone exposure- Vascularization- Decreased osteonecrosis and increased osteoclast density	[42]
Mouse	Subcutaneous administration of ZA and intraperitoneal injections of CY	Noncultured SVF	Tail-vein injection	- Reduced MRONJ lesion- Increased osteoblast and osteocytes number- Decreased necrotic bone area	[43]
Rat	Subcutaneous administration of ZA and dexamethasone	Allogenic BM-MSCs	Tissue-engineered cell sheet	- Active neoangiogenesis- Complete wound healing	[37]
Rabbit	IV ZA and dexamethasone	ADSCs	Direct placement into the extraction socket	- Gingival wound healing- Bone remodeling- Higher expression of TGF-β1 and fibronectin	[39]
Rat	Intraperitoneal ZA injection	ADSCs	Absorbable hemostatic gelatin sponge	- New bone formation- Intense vascularization	[44]
Rat	IV ZA	Allogenic BM-MSCs	Synthetic β-tricalcium phosphate (β-TCP) construct	- New bone formation, increased number of osteoclasts and osteoblasts	[45]
Mouse	Intraperitoneal ZA injection	ADSCs	Intravenous infusion	- Faster gingival epithelium healing, less bone exposure- New bone formation- Activation of autophagic flux	[38]
Rat	Subcutaneous administration of zoledronate and dexamethasone	Allogeneic BM-MSCs	Tissue-engineered cell sheet	- New bone laminar formation	[46]

**Table 2:** Overview of MSC-based treatment of MRONJ patients—case reports.

Patient’s Gender/Age	Primary Disease	Medication	Type of Stem Cells	Carrier of MSCs	Outcome	Ref.
Female/75	Severe osteoporosis	Alendronate and pamidronate	Autologous BM-MSCs	Fibrine sponge	- Bone healing; concentric ossification- Complete MRONJ healing	[60]
5 Females/54–771 Male/60	Breast cancer; osteoporosis	Oral alendronate and IV ZA	Autologous BM-MSCs	Collagen membrane	- Complete mucosal healing- No signs of MRONJ recurrences	[61]
Male/68	Multiple myeloma and bone disease	Cyclophosphamide, bortezomib, and dexamethasone; iv ZA	Autologous BM-MSCs	Bone substitute (Geistlich Bio-OssVR Collagen)	- Complete wound healing and bone regeneration- No pain or infection	[62]
Female/66	Metastatic breast cancer	IV ZA	Autologous BM-MSCs	Direct injection	- Almost complete bone regeneration- No pain or swelling	

## Data Availability

Not applicable.

## Abbreviations

The following abbreviations are used in this manuscript:

ONJ	Osteonecrosis of the jaw
MSC	Mesenchymal stem cells
BM	Bone marrow
RANK-L	Receptor activator of nuclear factor kappa-B ligand
MRONJ	Medication-related osteonecrosis of the jaw
BP	Bisphosphonate
ZA	Zoledronic acid
β-TCP	Β-tricalcium phosphate
IV	Intravenous infusion
miRNAs	MicroRNAs
lncRNAs	Long non-coding RNAs
ECM	Extracellular matrix
ADSC	Adipose tissue-derived stem cell
